# How Much Do Adolescents Cybergossip? Scale Development and Validation in Spain and Colombia

**DOI:** 10.3389/fpsyg.2018.00126

**Published:** 2018-02-12

**Authors:** Eva M. Romera, Mauricio Herrera-López, José A. Casas, Rosario Ortega Ruiz, Rosario Del Rey

**Affiliations:** ^1^Department of Psychology, Universidad de Córdoba, Córdoba, Spain; ^2^Department of Psychology, Universidad de Nariño, San Juan de Pasto, Colombia; ^3^Department of Psychology, Social Work and Counselling, University of Greenwich, London, United Kingdom; ^4^Department of Development and Educational Psychology, Universidad de Sevilla, Seville, Spain

**Keywords:** gossip, cybergossip, adolescence, self-report, psychometric properties, gender, cross-cultural

## Abstract

Cybergossip is the act of two or more people making evaluative comments via digital devices about somebody who is not present. This cyberbehavior affects the social group in which it occurs and can either promote or hinder peer relationships. Scientific studies that assess the nature of this emerging and interactive behavior in the virtual world are limited. Some research on traditional gossip has identified it as an inherent and defining element of indirect relational aggression. This paper adopts and argues for a wider definition of gossip that includes positive comments and motivations. This work also suggests that cybergossip has to be measured independently from traditional gossip due to key differences when it occurs through ICT. This paper presents the Colombian and Spanish validation of the *Cybergossip Questionnaire for Adolescents* (CGQ-A), involving 3,747 high school students (*M* = 13.98 years old, *SD* = 1.69; 48.5% male), of which 1,931 were Colombian and 1,816 were Spanish. Test models derived from item response theory, confirmatory factor analysis, content validation, and multi-group analysis were run on the full sample and subsamples for each country and both genders. The obtained optimal fit and psychometric properties confirm the robustness and suitability of a one-dimensional structure for the cybergossip instrument. The multi-group analysis shows that the cybergossip construct is understood similarly in both countries and between girls and boys. The composite reliability ratifies convergent and divergent validity of the scale. Descriptive results show that Colombian adolescents gossip less than their Spanish counterparts and that boys and girls use cybergossip to the same extent. As a conclusion, this study confirmes the relationship between cybergossip and cyberbullying, but it also supports a focus on positive cybergossip in psychoeducational interventions to build positive virtual relationships and prevent risky cyberbehaviors.

## Introduction

### Gossip

Gossip is defined as a type of evaluative conversation about somone else who is not present (Eder and Enke, 1991; Foster, 2004). These conversations have a situational nature, as the message emission and interpretation depend on the context in which they are produced (Wert and Salovey, 2004). Gossip is a widespread and repeated practice, to which a great part of daily conversations is usually devoted (Dunbar et al., 1997; Levin and Arluke, 2013). Gossip has and had a bad reputation throughout time and across cultures because it is considered as self-serving and seen to be carried out to maliciously manipulate the impressions the hearers have about a third person (Goodman and Ben-Ze'ev, 1994). Nevertheless, some evidence exists of the positive effects of gossip (Baumeister et al., 2004; Dunbar, 2004; Alexandrov et al., 2013).

Research on the positive side of gossip has mainly focused on its group-serving functions. It has been said that it can be useful to inform members of the group about its norms and sanctions (Grosser et al., 2010), to protect the group from those who break the rules (Beersma and Van Kleef, 2011; Feinberg et al., 2014), to influence its members' reputation (Giardini, 2012) and to strengthen social links within the group (Dunbar, 2004). Seen this way, gossip promotes the common good (Beersma and Van Kleef, 2012), and constrains self-serving behaviors that could damage the group given the influence it may have on members' reputation and inclusion (Beersma and Van Kleef, 2011). Individual benefits have also been ascribed to those who receive gossip, as it allows obtaining evaluative information about others that can serve as a reference with which to evaluate oneself (Wert and Salovey, 2004). In particular, gossip has been shown to increase one's self-improvement value, an important dimension of self-evaluation (Martinescu et al., 2014). This benefit reflects the learning function of gossip, promoting important social learning about how to achieve success or avoid failure in specific social situations (Baumeister et al., 2004). In this context, positive gossip is valuable to the individual because it provides a model to imitate (Litman and Pezzo, 2005). The advances in this line direct toward the necessity of making the gossipers' moral deliberation explicit to regulate the relationships and generate a greater confidence (Peters and Kashima, 2015).

In addition to providing information, influence, and strengthen group bonds, gossip also has a gratifying individual function: entertainment (Foster, 2004). What differentiates gossip from social curiosity—a related type of behavior—is its objective. While gossip predominantly has an entertainment purpose, social curiosity is mainly directed at obtaining information about other people (Hartung and Renner, 2013).

### Gossip, cybergossip, and cyberbullying

A recent study with more than 55,000 adolescents from 20 different countries shows the widespread use of ICT in social communication and its importance in social and personal development (Areepattamannil and Khine, 2017). Although there is general awareness of ICT's importance in adolescent relationships, the number of studies on cybergossip is limited (Oluwole, 2009; Laghi et al., 2013; Subramanian, 2013). Moreover, most of the studies that do exist have taken place within the field of computer science (e.g., Apolloni et al., 2013), and only a few have come forth from the psychology sphere (e.g., Gabriels and De Backer, 2016).

Cybergossip is the equivalent of gossip in the virtual world, but both phenomena must be studied in different ways, as the contexts in which they take place differ and matter. First, face-to-face gossip makes use of verbal language, whilst cybergossip can take the form of written messages, images, and videos. Second, cybergossip messages are recorded and can be reproduced exactly as originally created, which may influence what is shared in the first place (Bertolotti and Magnani, 2013). Third, cybergossip has the potential to reach a wide audience, at any time, and instantaneously. Fourth, given the limited prosodic, paralinguistic, and extralinguistic elements that can be used in virtual communication, it is harder for both the sender to convey the social intention behind the message and for the recipient to recognize it (Carrera and Pelayo, 2002). This increases the possibility of misunderstandings, which can lead to cyberaggression and cybervictimizing behaviors and therefore to cyberbullying (Del Rey et al., 2015).

It is also important to differentiate between cybergossip and cyberbullying. Gossip's negative potential has led numerous studies to identify it as an indirect form of peer group aggression that has the aim to manipulate another's reputation or achieve his/her exclusion from the group (Archer and Coyne, 2005). Nevertheless, a clear intention of damaging the other is not necessarily present in cybergossip, while it is in cyberbullying (Tokunaga, 2010). Therefore, while related, gossip, cybergossip, and cyberbullying are not directly comparable and must be studied with tools appropriately designed for each of these phenomena.

### The need for cybergossip scales for adolescents

Despite the differences between the phenomena, work on cybergossip should take research into its off-line version as a starting point. Gossip has been widely studied and with different research methodologies. As anthropology is the dominant field for the study of gossip, observational techniques have been most commonly used (Foster, 2004), but this is problematic. Confidence and discretion are two factors that influence gossip (Gluckman, 1963) and these conditions are difficult to guarantee in experimental and observational studies. Self-report studies do not violate these conditions due to their anonymous and retrospective nature, and therefore, despite drawbacks which are mainly related to the risk of obtaining socially desirable answers, form the ideal methodology for the measurement of gossip (Brady et al., 2017). While the need for such quantitative studies has been identified (Noon and Delbridge, 1993), to date, very few exist, and the scarce scales that are available have a number of limitations. Several scales too narrowly define gossip as a negative behavior and even as an indirect mode of aggression. Examples are the *Direct and Indirect Aggression Scale* (*DIAS*) (Björkqvist et al., 1992) and the *Indirect/Social/Relational Aggression* scale (*ISRA*) (Coyne et al., 2006). In these examples gossip is equated with expanding rumors and measured using only one item (commenting badly about others). Other instruments that do define gossip more widely, including both the positive and the negative potential, focus on specific aspects, such as the content of the message (Nevo et al., 1993) attitudes toward gossip (Litman and Pezzo, 2005), motivations behind gossip (Beersma and Van Kleef, 2012), and functions of gossip (Foster, 2004), but they do not measure the frequency of the behavior itself (Brady et al., 2017). A final limitation of the latter group of scales is the lack of European and Latin American samples used in their validation. Below, several existing instruments are described in a bit more detail.

The widely known *Tendency Gossip Questionnaire* (Nevo et al., 1993) was validated in a study with 120 Israeli college students. It consists of a 7-point frequency scale of 19 items that assess the content of conversations (physical appearance, achievement, social information, and sublimated gossip). This scale has been criticized for having too few items with which to measure the wide content variability possible in gossip (Litman and Pezzo, 2005).

The *Gossip Functions Questionnaire* (Foster, 2004) measures agreement with 24 statements related to the four basic functions that gossip is said to have: information provision, creation of friendship, attainment of influence, and entertainment. More research focussing on these functions has subsequently taken place with English speaking countries (USA, Canada, Australia, United Kingdom, Ireland and New Zealand) (Watson, 2011; Hartung and Renner, 2013), but both the original scale and the subsequent work fail to consider that gossip is subject to the context in which it happens (Paine, 1967) and that it is not a state (trait) or aptitude.

The *Motives to Gossip Questionnaire* (Beersma and Van Kleef, 2012), validated with university students in the Netherlands, does include context to some extent: 22 statements referring to specific behaviors and grouped in four dimensions (inform, entertain, influence, and meeting the norms of the group) were presented to undergraduate students, and the reasons for getting involved in their latest gossip experience were assessed. The drawback of this instrument is that it only assesses the respondents' latest gossip event and not the frequency of the behavior. Another criticism is that its influence dimension is solely negative, being related to indirect aggression. Stirling's (1956) and Foster's (2004) original studies recognize there can also be a positive side to the influence dimension, which is related to the learning of the group's culture and norms.

Brady et al. (2017) recently validated the *Workplace Gossip* scale with work-experienced Canadian students, which does include the frequency with which gossip is carried out and thereby claim this study helps resolve the lack of generality of the construct and the possible measurement bias from which previous studies suffered. While this is indeed a good step forward, its focus on the work environment, with its specific issues and dimensions, mean it might not be as relevant for other contexts.

The scales discussed above have all been designed and validated for (young) adults. For children and adolescents, gossip research exists mainly in the form of narrative and observational studies, and these approach gossip from the same narrow negative angle of indirect agression aimed at damaging others (Xie et al., 2005) as some of the studies that focus on adults. Results from evolutive studies show that as boys and girls grow up, they increasinly demonstrate indirect aggressive behaviors, such as gossip, to victimize their peers (Archer and Coyne, 2005). In ethnographic studies, adolescent girls are said to gossip more than boys with the aim to keep their social status (Merten, 1997). This view on the relationship between gender and gossip—understood in its malignant version—is confirmed in a review by McAndrew (2014). On the other hand, meta-analyses on gender differences in indirect aggression, including gossip, do not find a consistent relationship (Archer, 2004; Card et al., 2008).

Research into children and adolescents needs to adopt the methodological advances that were made in the study of gossip among adults, and include its positive potential to transmit understanding of group norms and see it as part of the set of socially competent behaviors (McDonald et al., 2007). It also needs to reflect the changes in interaction and social relationships brought about by ICT (Lee et al., 2016). Examples of scales that have focussed on online behaviors among adolescents and that cover cybergossip are the *Cyber-aggression Questionnaire for Adolescents* (CYBA) (Álvarez-García et al., 2016) and the *European Cyberbullying Intervention Project Questionnaire* (ECIPQ) (Del Rey et al., 2015). However, these suffer from the same limitation as some of the off-line gossip scales, in that they treat cybergossip as a form of cyberaggression and do not include positive forms of gossip. A focus on gossip in its wide sense is especially relevant in adolescence, because at this stage relationship styles are consolidated. Identifying and describing the behaviors that characterize these relationship styles is of great research interest because they predict important aspects of social and personal functioning in adulthood (Perry and Pauletti, 2011). This argument is strengthened by findings from Engelmann et al. (2016), who observed positive effects of gossip already in childhood.

To provide in the need for a cybergossip instrument for children the *Cybergossip Questionnaire-Primary* scale was designed and validated for schoolchildren aged 10–12 (López-Pradas et al., 2017). It was designed to meet the abovementioned needs relating to measurement, definition and the role of ICT. The instrument includes nine types of behaviors covering the four functions of gossip (influence, entertainment, information, and friendship). Validation showed the scale has the optimal psychometric properties to study cybergossip among primary school children, but, to date, no such scale exists for adolescents.

### The present study

The first objective of this study was to validate the *Cybergossip Questionnaire-Primary* scale (López-Pradas et al., 2017) for use among adolescents as well. A separate cyberbullying scale was used to analyse the discriminant validation of the cybergossip scale. The study used samples from two countries in different regions, Spain and Colombia, that have cultural similarities, a shared history and language. This ties it into the current trend of carrying out cross-cultural comparative studies, but adds diversity as, to date, these comparisons have most commonly taken place between European countries or between the United States and different Asian countries (Romera et al., 2017). The second objective was to measure the frequency of adolescents' use of cybergossip, and to analyze the possible differences by country of origin and gender. This validated scale will allow future research to gain further insights into de norms, values, conventions and behaviors of this age group when using ICT (Romera et al., 2016).

Our hypotheses were: (1) the instrument will show a one-factor structure with optimal psychometric properties, homogeneity of measure across gender and country and will have a low association with cyberbullying; (2) Colombian adolescents will be less involved in cybergossip, due to their lower use of ICT (Said-Hung, 2014); and (3) there will be no gender differences in the frequency of cybergossip (Card et al., 2008).

## Materials and methods

### Participants

The overall sample comprised 3,747 adolescents (48.5% boys) aged 10 to 19 (*M* = 13.98; *SD* = 1.69). The Colombian subsample comprised 1,931 students (46.9% boys), aged 10–19 (*M* = 14.22; *SD* = 1.89), and the Spanish subsample comprised 1,816 students (50.2% boys) aged 12–19 (*M* = 13.69; *SD* = 1.35). The study used a convenience sample based on accessibility.

### Instruments

#### Cybergossip questionnaire-adolescents (CGQ-A)

To measure cybergossip in adolescents, the GCQ-P scale designed for primary school children was used unchanged (López-Pradas et al., 2017). The instrument, which comprises nine items, is based around the four main functions of gossip: to inform (“Le cuento a mis amigos por las Redes Sociales o WhatsApp las cosas que me entero que les pasan a otros”—“I use social networks or WhatsApp to share stories I hear about others with my friends”); to influence (“He hablado sobre un compañero o amigo por las Redes Sociales o WhatsApp para que el grupo cambie su opinión sobre él”—“I have told things about a classmate or friend on social networks or WhatsApp to make the group change their opinion about him/her”); to create friendship (“Hablo sobre los demás en las redes sociales o WhatsApp porque me hace sentir más cerca de mi grupo de amigos o amigas”—“I talk about others on social networks or WhatsApp because it makes me feel closer to my group of friends”); and to entertain oneself (“Hablo con mi grupo de amigos de las Redes Sociales o WhatsApp sobre lo que les pasa a otros compañeros del colegio para divertirme”—“I talk with my friends on social networks or WhatsApp about what's going on with other classmates for fun”). Each of the nine items has a frequency Likert scale with the following values: 0 = “nunca”—*never*; 1 = “casi nunca”—*hardly ever*; 2 = “normalmente”—*averagely*; 3 = “casi siempre”—*almost always*; and 4 = “siempre”—*always*. The internal consistency of the one-dimensional scale was optimal (Ω_CGQ−P_ = 0.83) when originally validated for 10–12 year olds. The questionnaire in Spanish is included in Appendix A in Supplementary Material.

#### European cyberbullying intervention project questionnaire (ECIPQ) (Del Rey et al., 2015)

This instrument had previously been validated for both Colombia (Herrera-López et al., 2017) and Spain (Ortega-Ruiz et al., 2016). It is composed of 22 Likert-type items (11 for cybervictimization and 11 for cyberaggression) with five answer options from 0 to 4: 0 = never; 1 = once or twice; 2 = once or twice a month; 3 = around once a week; 4 = more than once a week. Examples of the items are: “Alguien me ha dicho insultos o groserías por internet”—someone has insulted me via internet; “He colgado vídeos o fotos compremetedoras de alguien en internet”—I have posted compromising videos or photos of someone on the internet. The internal consistency when validated originally was optimal: α_cybervictimization_ = 0.84, α_cyberaggression_ = 0.81, α_total_ = 0.87.

### Procedure

The research had a transversal, restrospective, ex post facto design, with one group and multiple measures (Montero and León, 2007). The study was approved by the Comité Coodinador de Ética de la Investigación Biomédica de Andalucía (Coordinating Ethics Committee of Biomedical Research of Andalusia) and was in accordance with all regulations concerning profesional ethics as stated in the International Conference on Harmonization Good Clinical Practice Guideline. The study was approved by the Spanish school boards and the Colombian schools' management, and written parental consent was obtained. In both countries, the students were visited and the anonymous, confidential and voluntary nature and the objective of the study were explained before the survey was taken. The authors were responsible for the data collection. Students had 20 min to fill in the questionnaire. The researchers were present during this time to answer any questions.

### Statistical analysis

Mardia's coefficient was calculated to assess the multivariate normality of the dataset using the program “R” (R Development Core Team, 2008) and package “MVN” (Kormaz et al., 2015).

The psychometric properties of the CGQ-A scale were verified through item response theory (IRT) analysis, calculating a three-parameter model (3PL) fitted to polytomous scales (Muraki, 1990). This approach gives values for each item on the parameters discrimation (a), difficulty (b) and probability of success or failure (c). This analysis was carried out using “eRm; colospace and mirt” libraries (version 3.3.2) in “R” (R Development Core Team, 2008).

To assess construct validity, confirmatory factorial analyses (CFA) were carried out using the EQS 6.2 program (Bentler and Wu, 2012). The maximum likelihood estimation method (MLE), robust scaling (Bryant and Satorra, 2012), and polychoric correlations (Morata-Ramírez and Holgado-Tello, 2013) were used, which is the recommended approach for categorical variables and under the absence of multivariate normality. The fit of the models was tested with the following indexes: Satorra-Bentler scaled chi square ($\chi_{\text{S}-\text{B}}^{2}$) (Satorra and Bentler, 2001); the comparative fit index (CFI) and the non-normality fit index (NNFI) (≥0.90 is adequate; ≥0.95 is optimal); the root mean square error of approximation (RMSEA) and the root mean square residual (SRMR) (≤0.08 is adequate; ≤0.05 is optimal) (Hu and Bentler, 1999). The Akaike information criterion (AIC) was used to compare the obtained models, where the best model has the lowest value.

The generalization of the model, that is, the degree of robustness of the factorial structure or invariance, was tested through multi-group analysis, with country and gender as analysis criteria. This analysis consists of comparing a set of increasingly restrictive models. In Model 1, configural invariance was tested by imposing the same factorial structure on both subsamples, and checking whether the fit indexes of the combined model indicated good model fit. Subsequently, in Model 2 the factorial loads were restricted and the fit indexes of Models 1 and 2 were compared. Changes (Δ) between the models of NNFI, CFI, RMSEA, and SRMR ≤0.01 were accepted as evidence of measurement invariance (Dimitrov, 2010). As a further test of invariance, the chi square difference test ($\Delta \chi_{\text{S}-\text{B}}^{2}$) was used, where non-significant differences demonstrate invariance in the models (Bollen, 1989; Satorra and Bentler, 2010). The multi-group analysis was carried out in EQS 6.2 (Bentler and Wu, 2012).

The discriminant validity was tested through Spearman's Rho correlations between the CGQ-A scale and the ECIPQ dimensions (cybervictimization and cyberaggression).

Internal consistency was analyzed with McDonald's Omega index (Ω), recommended for categorical variables under the absence of multivariate normality (Elousa-Oliden and Zumbo, 2008). For this we used the 9.2 Factor program (Lorenzo-Seva and Ferrando, 2006). The composite reliability (CR) was determined to test general reliability of the set of items, and we looked at the average variance extracted (AVE) to test the accuracy with which the construct is measured. The used cut-off values were 0.70 for CR and 0.50 for AVE (Hair et al., 2005).

The level of involvement in cybergossip was calculated by taking the average of the nine items. The adopted statistical significance level was 0.05.

## Results

### Psychometric properties of the cybergossip questionnaire for adolescents (CGQ-A)

Mardia's analysis generated a coefficient of skewness of 63.027; *p* > 0.05 and a coefficient of kurtosis of 279.537; *p* > 0.05, indicating the data fail to meet the assumption of multivariate normality.

The 3PL (IRT) analysis provided discrimination values above 1, which are considered good values; the difficulty degree of the items ranged from 0.05 to 1.60, which is acceptable (acceptable values range from −3 to 3); and the probability of failure values were low, indicative of high quality items (Baker, 1992) (see Table 1).

**Table 1:** Mean, standard deviation, skewness, kurtosis, and 3PL analysis (IRT).

**Items (CGQ-A)**		***M***	***SD***	**Skew**	**Kurt**	**a**	**b**	**c**
CG 1	I have made comments about other friends or classmates to get into a group on social networks or WhatsApp.	1.19	0.55	3.81	17.87	2.03	1.61	0.02
CG 2	I talk about others on social networks or WhatsApp because it makes me feel closer to my group of friends	1.41	0.83	2.43	6.20	1.89	1.00	0.04
CG 3	I have told things about a classmate or friend on social networks or WhatsApp to make the group change their opinion about him/her	1.45	0.83	2.10	4.50	1.92	1.00	0.08
CG 4	When I'm angry with a classmate or friend, I talk about it on social networks or WhatsApp	1.39	0.78	2.36	5.80	1.73	0.97	0.00
CG 5	I have said negative things about another person on social networks or WhatsApp without realizing it	1.40	0.74	2.24	5.72	1.99	0.76	0.00
CG 6	I have shared a classmate's secret with others on social networks or WhatsApp	1.29	0.68	2.90	9.58	1.76	1.19	0.00
CG 7	I use social networks or WhatsApp to share stories I hear about others with my friends	1.56	0.94	1.86	3.13	2.19	0.52	0.00
CG 8	When somebody in my group does something bad, I tell the rest of my classmates via social networks or WhatsApp so they know about it	1.33	0.71	2.61	7.67	2.23	0.99	0.02
CG 9	I talk with my friends on social networks or WhatsApp about what's going on with other classmates for fun	1.32	0.71	2.77	8.65	1.95	1.03	0.00

*a, discrimination; b, difficulty; c, probability of failure (random)*.

The inter-item correlation analysis showed values in the upper-middle range. The correlations between the CGQ-A scale and the ECIPQ cyberaggression and cybervictimization dimensions were in the medium range, suggesting a low collinearity and discriminant validity between the instruments (see Tables 2, 3).

**Table 2:** Matrix of CGQ-A polychoric correlations.

**Item**	**1**	**2**	**3**	**4**	**5**	**6**	**7**	**8**	**9**
1	1								
2	0.61	1							
3	0.54	0.52	1						
4	0.43	0.43	0.43	1					
5	0.46	0.47	0.45	0.63	1				
6	0.48	0.41	0.43	0.53	0.59	1			
7	0.46	0.43	0.53	0.58	0.59	0.61	1		
8	0.48	0.49	0.50	0.52	0.56	0.54	0.59	1	
9	0.49	0.49	0.43	0.49	0.56	0.53	0.58	0.59	1

*All correlations were significative, p < 0.01*.

**Table 3:** Spearman's Rho correlations between ECIPQ and CGQ-A.

**Factor**	**n (3,747)**		**1**.	**2**.	**3**.	**Skew**.	**Kurt**.
	***M***	***D.T*.**					
1. Cyberaggression	1.12	0.26	–			5.13	41.75
2. Cybervictimization	1.22	0.35	0.49^**^	–		3.85	23.06
3. Cybergossip	1.37	0.50	0.44^**^	0.30^**^	–	2.36	7.94

** *p < 0.01*.

**Figure 1:**
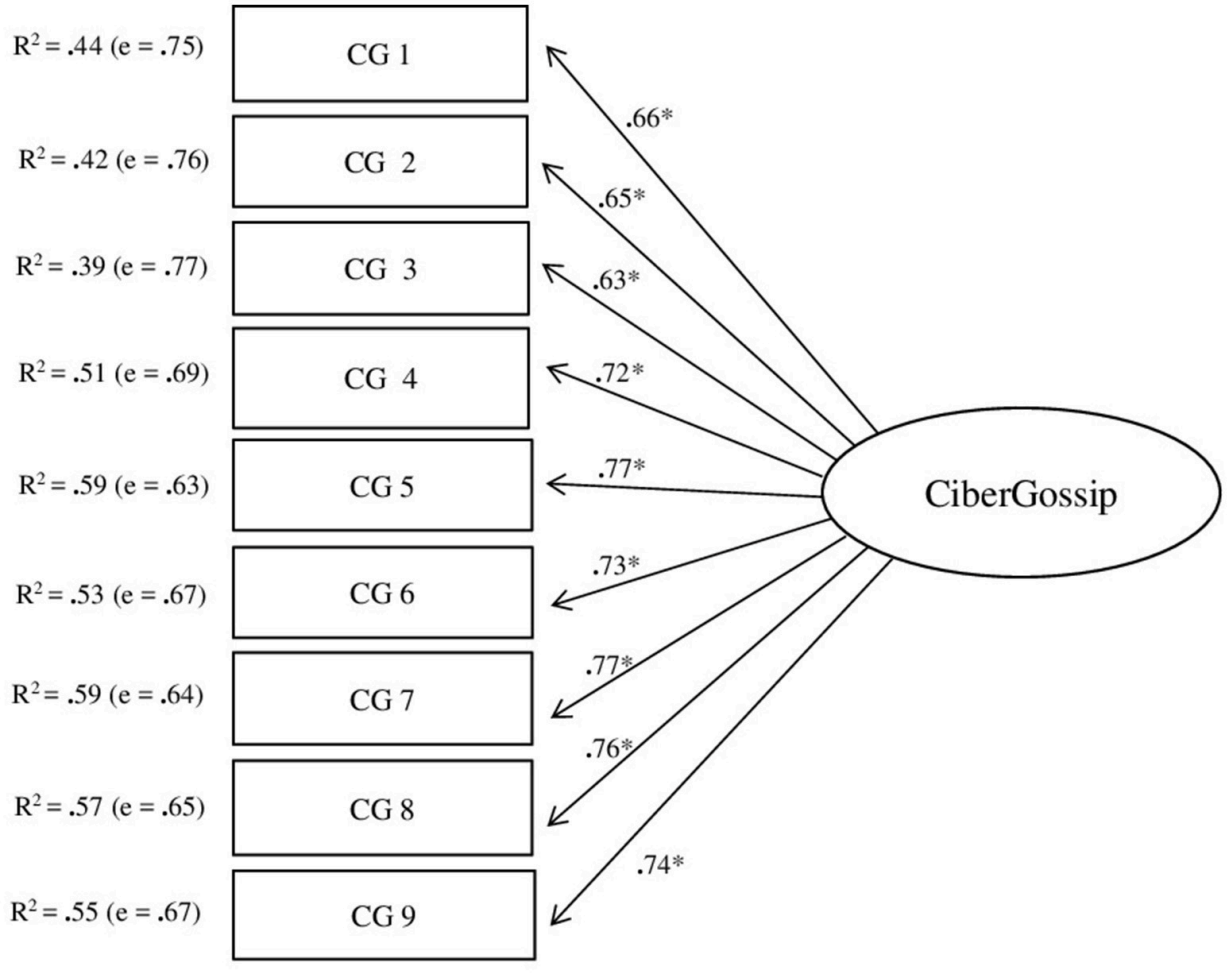
CFA of the CGQ-A scale (^*^p < 0.05).

The CFA of the one-dimension structure of the CGQ-A scale showed an adequate fit: $\chi_{\text{S}-\text{B}}^{2}$ = 286.581; *df* = 27; *p* < 0.001; NNFI = 0.979; CFI = 0.984; RMSEA = 0.055 (90% CI [0.050, 0.061]); SRMR = 0.048; AIC = 232.581 (see Figure 1).

The obtained value of the internal consistency was within the optimal range (Ω_CGQ−A_ = 0.839).

The values of the CR and AVE indexes were also found within the optimal range: CR = 0.904; AVE = 0.513, over 0.70 y 0.50, respectively (Hair et al., 2005).

The results of the multi-group analysis indicated proof of configural and measurement invariance for both the country and gender tests. For both variables, the chi-square differences ($\chi_{\text{S}-\text{B}}^{2}$) between Models 1 and 2 were non-significant and the deltas (Δ) of the CFI, NNFI, RMSEA and SRMR indexes in all comparisons were below the 0.01 cut-off point (see Table 4).

**Table 4:** Multi-group analysis of the CGQ-A scale per country and gender.

**Criterion**	**Mod**	**$\chi_{\text{S}-\text{B}}^{2}$**	***df***	***p***	**NNFI**	**CFI**	**RMSEA**	**SRMR**	**Δ $\chi_{\text{S}-\text{B}}^{2}$**	**Δ*p***	**Δ*df***	**ΔNNFI**	**ΔCFI**	**ΔRMSEA**	**ΔSRMR**
Country	Mod 1	287.072	54	0.000	0.966	0.975	0.053	0.061							
	Mod 2	276.523	62	0.000	0.973	0.977	0.047	0.061	10.549	0.534 (n.s.)	8	0.007	0.002	0.006	0.000
Gender	Mod 1	306.722	54	0.000	0.980	0.985	0.055	0.049							
	Mod 2	317.516	62	0.000	0.982	0.985	0.051	0.050	10.794	0.793 (n.s.)	8	0.002	0.000	0.004	0.001

*Mod 1, model without restrictions; Mod 2, model restricted on factor weights; n.s., non-significant*.

### Occurence of cybergossip among adolescents

The adolescent participants in the study had an overall cybergossip involvement level of 1.36 (*SD* = 0.49). Cybergossip occurred more often among the Spanish participants (*M* = 1.57; *SD* = 0.59) than the Colombian ones (*M* = 1.24; *SD* = 0.37) (*t* = 17.48; *p* > 0.001). As also hypothesized, no differences were found between girls and boys (*t* = 1.75; *p* = 0.08; *M* = 1.35; *SD* = 0.47 vs. *M* = 1.38; *SD* = 0.53, respectively).

## Discussion

The objective of the study was to validate the CGQ-P scale (López-Pradas et al., 2017) for Spanish and Colombian adolescents (afterwards called *Cybergossip Questionnaire-Adolescents*, CGQ-A). The results confirmed the original one-dimensional structure with optimal indexes of fit and internal consistency. The results of the discriminant validity analysis showed that the CGQ-A scale measures a different concept than the cyberaggression and cybervictimization dimensions of the ECIPQ scale, although they are related. These results underline the problem of a narrow conceptualization of gossip as malicious behavior, and support taking a wide perspective that encompasses positive aspects of social learning (Wert and Salovey, 2004; Beersma and Van Kleef, 2012).

The multi-group analysis proved configural and measurement invariance between Colombia and Spain and between boys and girls, indicating the scale has a robust factorial structure across countries and gender. Put in another way, it means the different items contribute similarly to the overall factor for girls and boys, for Columbians and Spanish. The invariance therefore demonstrates that Colombian and Spanish adolescents conceptualize cybergossip in similar ways when they respond to the CGQ-A scale. This indicates similar dynamics in both countries, despite the cultural, socioeconomic and geographical differences (Romera et al., 2017). Although gossip has been widely identified as a universal behavior (Dunbar, 2004), this study is the first to analyze this in Spanish and Colombian samples, and to show that, also among adolescents and when carried out via ICT, gossip has a cross-cultural nature. The invariance results also demonstrated a shared conceptualization between boys and girls. This contradicts some earlier research that classified gossip as a form of indirect aggression, and found that girls, to a greater extent than boys, see gossip as a form of aggression (Archer and Coyne, 2005). Our results are in line with research on gossip in a wide sense, which shows it is a behavior well-recognized and identified with by not only girls but also boys (Kuttler et al., 2002).

The second objective of the research was to measure how frequenctly adolescents engage in cybergossip, and to investigate differences by country and gender. The descriptive results indicate that they enage in this cyberbehavior quite frequently. Results confirmed our second hypothesis that Colombian adolescents would be less involved in cybergossip than Spanish ones. This could reflect the lower use of ICT in Colombia (Said-Hung, 2014), but could also be due to differences in culture. Spanish culture is characterized by a greater promotion of individualist values when it comes to social image and recognition and acceptance within the group (Tafarodi and Swann, 1996), which would be in line with greater involvement in this interactive phenomenon. On the other hand, Latin American culture is more restrictive, has a higher respect for rules, and approval and obedience are valued more (Lila et al., 2000), which could explain a lower involvement in behaviors that are commonly seen as negative.

No differences were found in the frequency with which boys and girls participate in cybergossip, which confirms the third hypothesis. This, together with the results on measurement invariance by gender, supports the idea that boys and girls engage in gossip in similar ways. Previous developmental studies have shown a higher involvement of girls, which they attributed to adolescent girls spending more time on social activities, and having a relationship style characterized by a desire for proximity and an anxiety about rejection (Perry and Pauletti, 2011). In the literature on indirect aggression girls have also been seen to gossip more (Archer and Coyne, 2005), and this relationship between gossip and gender was found in studies on adults as well (Nevo et al., 1993). Nevertheless, our results are in line with the majority of contributions in this field, regardless of whether studies with a wide definition (Foster, 2004) or a narrow one (Kuttler et al., 2002; Card et al., 2008) are considered. This leads to the question why there is a false stereotyped view that girls are bigger gossipers than boys (in both the narrow and wide sense of the concept). Some studies attribute this association to gender schemas, built since childhood, which determine the processing of social information (Card et al., 2008). Another conclusion from these results should be that it is essential to include boys in the study on gossip and cybergossip, unlike several studies in the past that used only female subjects (McDonald et al., 2007; Massar et al., 2012).

## Conclusions

The present study has made an important contribution to this field of research by providing it with a valid and reliable instrument with which to measure cybergossip behavior. The CGQ-A scale's optimal psychometric properties and general validity allow its use in comparative studies of descriptive and explanatory nature.

Without downplaying the possible harmful effects of gossip, this paper argues that cybergossip does not have to be understood as a cyberbehavior which must be erradicated or reduced in all situations. This research recognizes and has confirmed the relationship between cybergossip and cyberbullying, but it also supports a focus on positive cybergossip in psychoeducational interventions that promote the learning of new methods of interaction and the development of communication and digital skills to build positive virtual relationships and prevent risky cyberbehaviors.

### Limitations

A number of limitations of this study must be mentioned. No explicit tests of convergent validity have been included. These were omitted as this study's particular aim was to observe its divergent validity, which is why we focussed on the correlation between the concepts. Another potential limitation is that no test-retest measures have been employed to confirm the reliability of the results, although different samples have been used and the optimal psychometric properties of the CGQ-A scale have been established.

### Future research

It is necessary to continue advancing our understanding of gossip, especially of its effect on group characteristics, following recent evolutionary studies that highlight the influence of social networking on social behavior (Wu et al., 2016). The role of gender in cybergossip also requires further attention, for example with respect to the possible nuances that relational variables such as friendship may introduce (Watson, 2012). Another interesting direction of research would be to follow up on the findings by Areepattamannil and Khine (2017) that personal (availability, ability, habit, uses) and motivational (self-efficacy, interest, enjoyment) factors correlate with the use of ICT for gossip. It is also necessary to generate new research of a developmental nature to better understand what is behind differences in involvement in gossip and cybergossip.

## Ethics statement

The study was approved by the Comité Coodinador de Ética de la Investigación Biomédica de Andalucía (Coordinating Ethics Committee of Biomedical Research of Andalusia) and was in accordance with all regulations concerning professional ethics as stated in the International Conference on Harmonization Good Clinical Practice Guideline. The study was approved by the Spanish school boards and the Colombian schools' management, and written parental consent was obtained. In both countries, the students were visited and the anonymous, confidential and voluntary nature and the objective of the study were explained before the survey was taken.

## Author contributions

All authors contributed to the interpretation of data, helped to draft, and revise the manuscript and have read and approved the final manuscript.

### Conflict of interest statement

The authors declare that the research was conducted in the absence of any commercial or financial relationships that could be construed as a potential conflict of interest.
