# Supplementary material for: How Much Do Adolescents Cybergossip? Scale Development and Validation in Spain and Colombia
Source: Front Psychol. 2018 Feb 12;9:126. doi: 10.3389/fpsyg.2018.00126 (PMC5816232; doi:10.3389/fpsyg.2018.00126)
Supplement: Supplementary file 1 [file Table1.pdf]

Supplementary Material

How much do adolescents cybergossip? Scale development and validation in Spain and Colombia

Romera, Eva M.*, Herrera-López, M., Casas, J. A., Ortega-Ruiz, R., Del Rey, R.

*** Correspondence:** Eva M. Romera: eva.romera@uco.es

1 Supplementary Material. Appendix A

Items *Cybergossip Questionnaire for Adolescents (CGQ-A).* Spanish version.

CG1. He hecho comentarios de otros amigos o compañeros para conseguir estar dentro de un grupo en las Redes Sociales o WhatsApp [I have made comments about other friends or classmates to get into a group on social network or WhatsApp].
CG2. Hablo sobre los demás por las Redes Sociales o WhatsApp porque me hace sentir más cerca de mi grupo de amigos o amigas [I talk about others on social networ or WhatsApp because it makes me feel closer to my group of friends].
CG3. He hablado sobre un compañero o amigo por las Redes Sociales o WhatsApp para que el grupo cambie su opinión sobre él o ella [I have told things about a classmate or friend on social network or WhatsApp to make the group change their opinion about him/her].
CG4. Cuando me enfado con un compañero o amigo lo cuento en las Redes Sociales o WhatsApp [When I'm angry with a classmate or friend, I talk about it on social network or WhatsApp].
CG5. He contado cosas malas sobre otra persona por las Redes Sociales o WhatsApp sin darme cuenta de ello [I have said negative things about another person on social network or WhatsApp without realizing it].
CG6. He contado un secreto que me ha dicho un compañero o compañera de clase por las Redes Sociales o WhatsApp [I have shared a classmate's secret with others on social network or WhatsApp].
CG7. Le cuento a mis amigos por las Redes Sociales o WhatsApp las cosas que me entero que les pasan a otros [I use social network or WhatsApp to share stories I hear about others with my friends].
CG8. Cuando alguien de mi grupo hace algo que está mal, lo cuento al resto de compañeros por las Redes Sociales o WhatsApp para que lo sepan [When somebody in

my group does something bad, I tell the rest of my classmates on social network or WhatsApp so they know about it].
CG9. Hablo en mi grupo de amigos de las Redes Sociales o WhatsApp sobre lo que les pasa a los otros compañeros del colegio para divertirme. [I talk with my friends on social network on or WhatsApp about what's going on with other classmates for fun].
